# Staphylococcal entertotoxins of the enterotoxin gene cluster (egcSEs) induce nitric oxide- and cytokine dependent tumor cell apoptosis in a broad panel of human tumor cells

**DOI:** 10.3389/fcimb.2013.00038

**Published:** 2013-08-13

**Authors:** David S. Terman, A. Serier, O. Dauwalder, C. Badiou, A. Dutour, D. Thomas, V. Brun, J. Bienvenu, J. Etienne, F. Vandenesch, G. Lina

**Affiliations:** ^1^Molecular Genetics Program, Jenomic Research InstituteCarmel, CA, USA; ^2^CIRI, International Center for Infectiology Research, LabEx Ecofect, Université Lyon1, Inserm U1111, Ecole Normale Supérieure de Lyon, CNRS UMR5308Lyon, France; ^3^Centre National de Références des Staphylocoques, Hospices Civils de LyonBron, France; ^4^Unité INSERM U590 équipe Cytokines et Cancer, Centre Léon BérardLyon, France; ^5^Laboratoire d'Etude de la Dynamique des Protéomes, U880 CEA/DSV/iRTSV/INSERM/UJFGrenoble Cedex, France; ^6^Laboratoire d'Immunologie, Centre de Biologie et Pathologie Sud, Hospices Civils de Lyon, Chemin du Grand RevoyetPierre Benite, France

**Keywords:** *Staphylococcus aureus*, egcSE superantigens, nitric oxide, tumor cell apoptosis

## Abstract

The egcSEs comprise five genetically linked staphylococcal enterotoxins, SEG, SEI, SElM, SElN, and SElO and two pseudotoxins which constitute an operon present in up to 80% of *Staphylococcus aureus* isolates. A preparation containing these proteins was recently used to treat advanced lung cancer with pleural effusion. We investigated the hypothesis that egcSEs induce nitric oxide (NO) and associated cytokine production and that these agents may be involved in tumoricidal effects against a broad panel of clinically relevant human tumor cells. Preliminary studies showed that egcSEs and SEA activated T cells (range: 11–25%) in a concentration dependent manner. Peripheral blood mononuclear cells (PBMCs) stimulated with equimolar quantities of egcSEs expressed NO synthase and generated robust levels of nitrite (range: 200–250 μM), a breakdown product of NO; this reaction was inhibited by NG-monomethyl-L-arginine (L-NMMA) (0.3 mM), an NO synthase antagonist. Cell free supernatants (CSFs) of all egcSE-stimulated PBMCs were also equally effective in inducing concentration dependent tumor cell apoptosis in a broad panel of human tumor cells. The latter effect was due in part to the generation of NO and TNF-α since it was significantly abolished by L-NMMA, anti-TNF-α antibodies, respectively, and a combination thereof. A hierarchy of tumor cell sensitivity to these CFSs was as follows: lung carcinoma > osteogenic sarcoma > melanoma > breast carcinoma >neuroblastoma. Notably, SEG induced robust activation of NO/TNFα-dependent tumor cell apoptosis comparable to the other egcSEs and SEA despite TNF-α and IFN-γ levels that were 2 and 8 fold lower, respectively, than the other egcSEs and SEA. Thus, egcSEs produced by *S. aureus* induce NO synthase and the increased NO formation together with TNF-α appear to contribute to egcSE-mediated apoptosis against a broad panel of human tumor cells.

## Introduction

*Staphylococcus aureus* produces a broad range of exoproteins, including staphylococcal enterotoxins and staphylococcal-like enterotoxins (SEs and SEls; respectively). To date, 23 different SEs have been described: they are designated SE A to X. All these toxins share superantigenic properties by stimulating a large proportion of T cells after binding to the major histocompatibility complex (MHC) class II molecule and crosslinking specific vβ regions of the T-cell receptor (TCR). This interaction results in polyclonal T-cell activation and massive secretion of cytokines such as interleukin-2 (IL)-2, interferon gamma (IFN-γ), tumor necrosis factor alpha (TNF-α), and nitric oxide (NO) (Marrack and Kappler, 1990). Several members of this group have been implicated in the pathogenesis of toxic shock syndrome and food poisoning, and have shown anti-tumor activity in animal models (Bohach, 2006; Terman et al., 2006). The egcSEs comprise five genetically linked staphylococcal enterotoxins, SEG, SEI, SElM, SElN and SElO and two pseudotoxins which constitute an operon present in up to 80% of *S. aureus* isolates (Jarraud et al., 2001; Becker et al., 2003). The egcSEs are structurally homologous and phylogenetically related to classic SEA-E and exhibit unique vβ signatures (Jarraud et al., 2001). Despite their prevalence and broad distribution, human serum levels of neutralizing antibodies directed against the egcSEs are significantly lower than those directed to the classic SEs (Holtfreter et al., 2004). This has been ascribed to defective mRNA transcription and impaired extracellular secretion (Grumann et al., 2008; Xu and McCormick, 2012). Interestingly, septicemia associated with the egcSEs has been reported to be less severe clinically than that linked to the classic SEs (Ferry et al., 2008).

Nitric Oxide (NO) is a pleiotropic molecule that mediates a broad spectrum of biologic functions including vasodilatation, neurotransmission, and immune defense (Moncada and Higgs, 1993; Bogdan, 2001). NO is produced by mammalian cells from one of the NG-guanidino nitrogens of L-arginine, in a reaction catalyzed by a NADPH-dependent dioxygenase and referred to as NO synthase (Kwon et al., 1990). The latter can exist in at least two distinct isoforms the first of which is a calcium-dependent NO synthase present mainly in neuronal cells (Bredt and Snyder, 1990) and vascular endothelial cells (Förstermann et al., 1991). The second enzyme is a calcium-independent inducible NO synthase found in macrophages (Marletta et al., 1988), hepatocytes (Billiar, 1990), endothelial cells (Radomski et al., 1990), and smooth muscle cells (Busse and Mülsch, 1990) after activation by bacterial lipopolysaccharide (LPS) or cytokines. NO from inducible NO synthase is responsible for killing microbial pathogens and tumor cells by activated macrophages (Hibbs et al., 1987, 1988; Nathan and Hibbs, 1991) and is further involved in the pathogenesis of LPS- or cytokine-induced hypotension and shock (Thiemermann and Vane, 1990). Tumor-associated NO, produced by tumor cells and/or host cells that permeate tumors, exerts both inhibitory and activating effects on carcinogenesis, tumor growth, angiogenesis, and metastases that appear to be concentration dependent. For example, activated macrophages and endothelial cells may produce cytotoxic levels of NO *in vitro* and prevent tumor growth and metastasis, presumably by killing tumor cells arrested or passaging through blood vessels or sinusoids (Hibbs et al., 1987; Xie and Fidler, 1998; Bogdan, 2001). Over-production of endogenous NO by tumor cells is auto-cytotoxic and suppresses tumor growth and metastasis (Hibbs et al., 1987; Xie and Fidler, 1998; Shi et al., 1999; Motterlini et al., 2000; Fukumura et al., 2006; Ma et al., 2010). Conversely, low levels of *NOS II* expression appear to promote tumor progression by interdicting tumor cell apoptosis (Billiar, 1990), altering blood vessel formation and tumor vasomotor tone (Busse and Mülsch, 1990; Radomski et al., 1990). The net effect of NO on tumor growth or apoptosis depends not only on its source and relative levels but also on the degree of activation of HIF1-α, heme oxygenase-1 (HO-1) and VEGF in the tumor microenvironment (Tamir and Tannenbaum, 1996; Ambs et al., 1997; Fukumura and Jain, 1998; Wink et al., 1998; Xie and Fidler, 1998).

Superantigens have been shown to induce tumor cell cytotoxicity *in vitro* and *in vivo* using several mechanisms. These include superantigen dependent cellular cytotoxicity (SDCC) wherein SAgs efficiently bind MHC class II-positive tumor cells and subsequently trigger human T cell proliferation and differentiation into cytotoxic T cells that kill tumor cells in a perforin/granzyme dependent manner (Dohlsten et al., 1995). In addition, MHCII deficient tumor cells are activated by selected SEs to express CD-54 which costimulates T cells in a vβ specific manner (Lamphear et al., 1998). T cell activation under these conditions may also be enhanced via a newly recognized B7- domain present in selected SEs which interfaces with T cell costimulatory receptor CD28 (Arad et al., 2011). Moreover, SAgs activated T cells and monocytes also produce various cytolytic cytokines notably IFN-γ, TNF-α, IL-2 which alone or together with nitrous oxide can induce cytotoxicity in both MHCII^+^ and MHCII- tumor cells (Fast et al., 1991; Dohlsten et al., 1993). Whereas SDCC is effective only against MHCII^+^ tumor cells (largely B cell lymphomas), cytokines and NO are able to kill MHCII^−^ carcinomas, fibrosarcomas and mastocytomas (Fast et al., 1991; Dohlsten et al., 1993, 1995).

Together with neutralizing antibodies against superantigens, the very same cytokines that mediate tumoricidal effects are considered to be the major factors underlying the constitutional and hemodynamic toxicity of the SEs *in vivo* (Bette et al., 1993; Miethke et al., 1993; Giantonio et al., 1997; Cheng et al., 2004). In the last decade, modification of SEA's MHCII binding affinity has led to reduced toxicity *in vivo* (Abrahmsen et al., 1995; Erlandsson et al., 2003). Newer versions of SEA devoid of epitopes for preexisting neutralizing antibodies are presently being tested in clinical trials against renal cell carcinoma. Importantly, naturally occurring antibodies against the egcSEs were found in sera of less than 5% of human sera compared to 50–80% for the classic SEs (Holtfreter et al., 2004).

Here, we examined the ability of egcSEs to generate tumoricidal molecules from PMBCs. In addition, we identified such products in cell free supernatants and investigated their ability to induce cytotoxicity against a broad panel of human tumor cells. These findings unveil an efficient NO and cytokine dependent tumor killing mechanism conserved in egcSEs in the presence of diverse levels of T cell activation and TH-1 cytokines.

## Materials and methods

### Preparation of recombinant SEs

SEA, SEG, SEI, SElM, SElN and SElO were produced in *Escherichia coli* M15 as His-tagged recombinant toxins and purified by affinity chromatography on a nickel affinity column according to the supplier's instructions (New England Biolabs, Ipswich, USA) as previously described (Thomas et al., 2009). Protein purity was verified by SDS-PAGE. LPS was removed from toxin solutions by affinity chromatography (Detoxi-GEL endotoxin Gel®, Pierce Rockford, USA). The QCL-1000 *Limulus* amebocyte lysate assay® (Cambrex-BioWhittaker, Walkersville, USA) showed that the endotoxin content of the recombinant SAg solutions was less than 0.005 units/mL.

### Tumor cells

Laryngeal squamous cell carcinoma cell line Hep-2 and human non-small cell lung adenocarcinoma CRL5800 were obtained from cell library (IFR128, Lyon, France). Osteogenic sarcoma CRL1547, human breast cancer cell line MDA-MB-549, human neuroblastoma cell line SK-N-BE and human melanoma PLA-OD were a gift from Raphael Rousseau (Centre Leon Berard, Lyon, France). They were cultured in DMEM (Gibco, Invitrogen Corporation, Cergy Pontoise, France) supplemented with 10% fetal calf serum (BioWest, Paris, France), 100 U/mL penicillin and 100 μg/mL streptomycin. For SK-N-BE cells, the medium was supplemented with 1% non-essential amino acids (Gibco, Invitrogen Corporation, Cergy Pontoise, France).

### Isolation of human mononuclear cells

Blood packs were obtained from healthy donors through a convention with Etablissement Français du Sang after a written informed consent and according to Declaration of Helsinki principles. Peripheral blood mononuclear cells (PBMCs) were isolated by Ficoll-Paque Plus® density gradient centrifugation (GE Healthcare Life Science, Orsay, France) and were washed with Ca- and Mg- free PBS. Cell viability was measured with the trypan blue exclusion test (>98%). The cells were washed in RPMI 1640 medium (Gibco, Invitrogen Corporation, Cergy Pontoise, France).

### T cell activation

T cell activation with various SEs was assayed by measuring surface CD69 expression. Briefly, PBMCs (10^6^ cells/mL) were incubated with SEA, SEG, SEI, SElM, SElN or SElO (1 pg/mL to 10 ng/mL) in Eagle's minimum essential medium (EMEN) containing 10% heat-inactivated FCS (Gibco Invitrogen, Paisley, UK) for 24 h at 37°C in humidified air with 5% CO_2_. EMEN and 100 μg/mL of phytohaemagglutinin (PHA) (Sigma-Aldrich, Saint Quentin Fallavier, France) were used as negative and positive control, respectively. Activated PBMCs were incubated with a mixture of anti-CD3 conjugated to cyanin-5-PE (Dako, Glostrup, Denmark) and anti-CD69 conjugated to PE (Beckman Coulter, Miami, FL). The cells were then analyzed with a FACScan® flow cytometer (BD Biosciences, San Jose, CA), and the results were expressed as the percentage of CD3+ lymphocytes expressing CD69.

### Cytotoxicity assays

3-(4,5-Dimethylthiazol-2-yl)-2,5-diphenyltetrazolium bromide (MTT) cytotoxic assay was performed to investigate the effect of stimulated PBMCs supernatants on cell viability (Mosmann, 2003). Tumor cells, 10^5^ cells/well, were seeded in 96-well plates and incubated with 10%, 20%, 50% or 100% of supernatants from SE-stimulated or unstimulated PBMCs. After 1–4 days, 10 μL of MTT solution (5 mg/mL) (Invitrogen Corporation, Cergy Pontoise, France) was added to culture wells and plates were incubated for 3 h at 37°C. Supernatant was removed and 100 μL of 0.04 N HCl in isopropanol was added to each well before reading optical density at 540 nm with an ELISA-Reader (Bio-Rad, Marne la Coquette, France). In some experiments, 100 μg/mL PHA was used as positive control for T cell activation. Cell toxicity data are expressed as percent of the mean value obtained for untreated cells.

### Annexin V-FITC/propidium iodide (PI) staining

Tumor cells, 10^5^ cells/well, were seeded in 96-well plates and incubated with 10% of SEs-stimulated PBMCs supernatant. After 4, 12, and 24 h, cells were harvested, washed with serum-containing medium and centrifuged at 3000 rpm for 5 min. The supernatant was discarded and the pellet was resuspended in 500 μL of 1× binding buffer. The sample solution was incubated with 1 μL of 5× FITC-conjugated annexin V (Abcam, Paris, France) and 1 μL of propidium iodide (PI) (Becton Dickinson, Le Pont de Claix, France) in the dark for 10 min at room temperature. The samples were analyzed using FACScanto II® flowcytometer (Becton Dickinson, Le Pont de Claix, France). Data analysis was performed with the FacsDiva® software (Becton Dickinson, Le Pont de Claix, France).

#### Nitric oxide (NO) assay

NO production was assessed by measuring the accumulated levels of nitrite in the supernatant with Griess reagent as previously described (Xie et al., 1995). Briefly, 100 μL of Griess reagent (1% sulfanilamide, 0.1% naphthylethylenediamine dihydrochloride, and 2.5% H_3_PO_4_) was added to 300 μL of the cell culture supernatant for 30 min at room temperature. Optical densities were read on a spectrophotometer at 548 nm. The values of NO concentration in the culture samples were obtained from standard curve of sodium nitrite solutions. For NO inhibition assays, PBMCs were incubated with or without a NO synthase inhibitor, L-NG-monomethyl arginine citrate (L-NMMA) (Sigma-Aldrich, Saint-Quentin Fallavier, France), for 2 h prior to stimulation by SEs (final concentration 3 mM). NO was quantified in supernatants as described above. Toxicity was analyzed by annexin-V and PI measurements as described above.

#### Cytokine assays

Levels of the cytokines IL-2, IL-4, IL-10, IL-12-p70, IL-17, IFN-γ, TNF-α, and Granulocyte Macrophage-Colony Stimulating Factor (GM-CSF) were measured in supernatants of PBMCs (10^6^ cells/mL) incubated with 1 pg/mL of SEA, SEG, SEI, SElM, SElN or SElO in EMEM containing 10% heat-inactivated FCS (Gibco Invitrogen, Paisley, UK) for 24 h at 37°C in humidified air with 5% CO_2_, using Milliplex® kits based on Luminex® technology (Millipore, Molsheim, France). EMEM and 100 μg/mL PHA were the negative and positive control, respectively.

#### Neutralizing antibodies against TNF-α

PBMC supernatants were incubated with and without 30 μg/mL of mouse neutralizing polyclonal anti-TNF-α antibody (Abcys, Paris, France) for 18 h at 22°C before carrying out the cytotoxic assays described above. Recombinant TNF-α, (Abcam, Paris, France), 50 ng/mL was used as positive control.

#### Statistical analysis

The statistical analyses were based on Student *t*-test or Wilcoxon test for non-parametric analysis. The level of statistical significance was set at 0.05. The tests were carried out with SPSS Statistics® version 19 software (IBM France, Bois Colombes, France).

## Results

### egcSEs induce T cell CD69 expression

**Figure 1:**
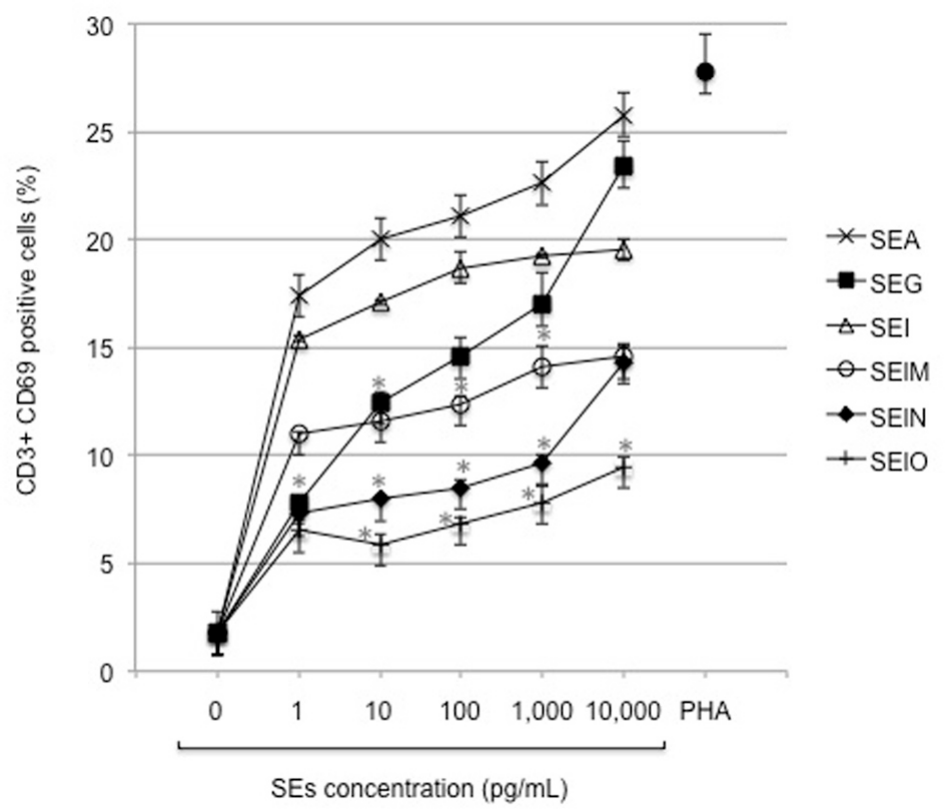
**Expression of early T cell surface marker CD69 after incubation of human PBMCs with SEA and egcSEs**. CD69 expression was measured on T lymphocytes (CD3+) after 24 h of incubation with PBMCs (10^6^ cells/mL) in presence of SEA, SEG, SEI, SElM, SElN, or SElO (0–10 ng/mL). PHA (100 μg/mL) was used as positive control. Results are shown as the mean ± SEM for each point (*n* = 3 independent experiments). Asterisks indicate statistical significance compared to SEA.

CD69, a C-type lectin, disulfide linked homodimer is the earliest T cell surface activation receptor appearing even before cytokine production (Sutkowski and Huber, 2003). It is activated by superantigens, cytokines, and PHA. Thus, we investigated the ability of SEA and our recombinant egcSEs to activate CD69 expression in T cells obtained from 3 healthy donors. We noted a hierarchy of T cell activation as follows: SEA > SEI >SEG>SEM>SEO>SEN. Overall, SEA activated a larger number of T cells (26%) than all of egcSEs (*p* = 0.005). Among the egcSEs, SEI and SEG were the most effective T cell stimulants, activating 19 and 23% of resting T cells respectively, levels that were significantly higher than the SEM, SEN, and SEO (*p* = 0.019) (Figure 1). These findings are consistent with the T cell activation by egcSEs demonstrated previously using a T cell proliferation assay (Grumann et al., 2008).

### Supernatants from egcSE-activated PBMCs kill a broad sampling of human tumor cells

**Figure 2:**
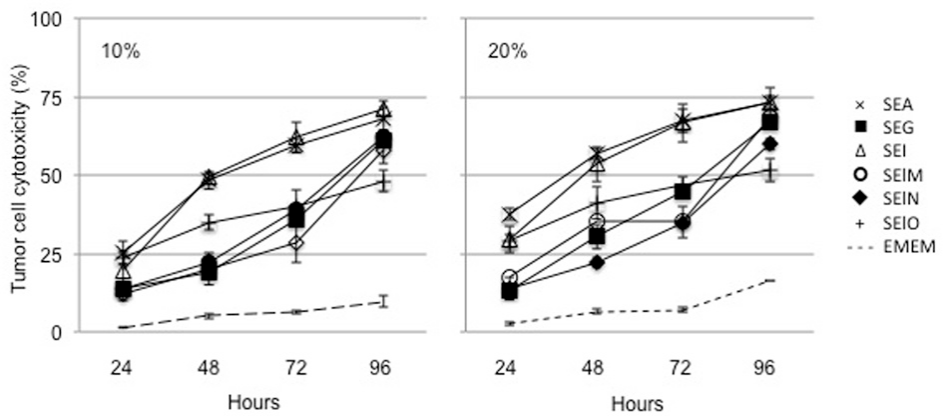
**Cytotoxicty of SEA and egcSE-stimulated PBMCs against Hep2 squamous cell carcinoma cells**. Supernatants from unstimulated PBMCs, SEA-, SEG-, SEI-, SElM-, SElN-, or SElO-stimulated PBMCs were added at 10% or 20% vol/vol to Hep2 squamous cell carcinoma cell culture. After 1–4 days, cell viability was evaluated by MTT asays. Results are shown as the mean ± SEM for each point (*n* = 3 independent experiments).

We examined the ability of supernatants from egcSE- and SEA-activated PBMCs to kill a broad sampling of human tumor cell lines. In preliminary experiments, Hep-2 squamous carcinoma cells were incubated for 96 h with various dilutions of supernatants of PBMCs that had been stimulated for 24–96 h with each egcSE, SEA or PHA. All supernatants from SE-stimulated PBMCs showed significant time and dose dependent cytotoxicity for each incubation time exceeding that of the control supernatant from unstimulated control PBMCs (*p* < 0.001) (Figure 2) (data 50% and 100% not shown). Supernatants from PBMCs incubated with SEs for 72 h consistently produced greater Hep-2 cell cytotoxicity than those supernatants similarly incubated for 48 h (*p* < 0.001). Thus, for subsequent experiments using other tumor cell lines we elected to use supernatants from PBMCs incubated with SEs for 72 hours.

**Figure 3:**
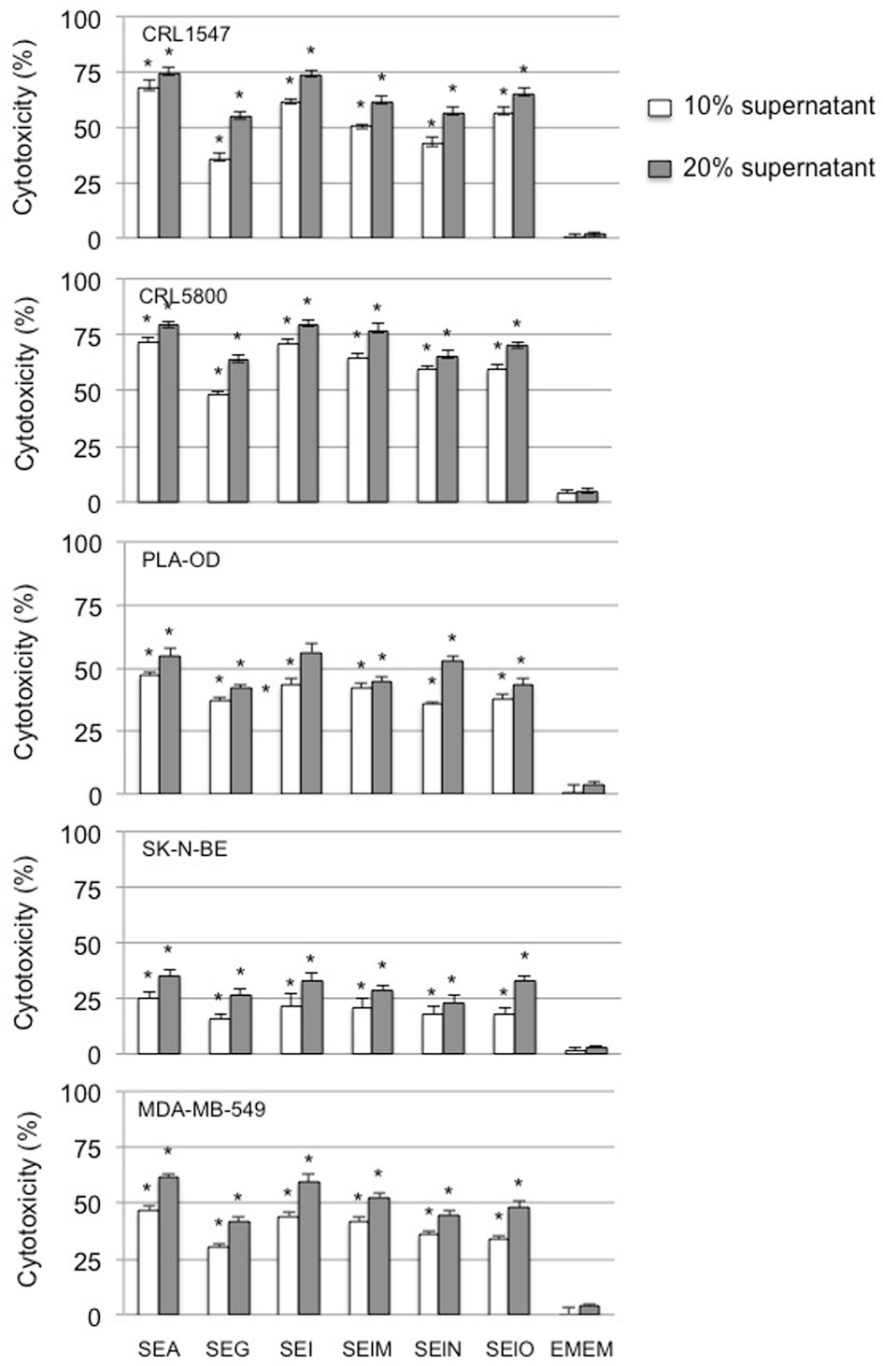
**Cytotoxicty of SEA and egcSE-stimulated PBMCs vs. a broad panel of human tumor cell lines is shown**. Cytotoxicity of 72 h supernatants from unstimulated PBMCs, SEA-, SEG-, SEI-, SElM-, SElN-, or SElO-stimulated PBMCs against human non-small cell lung adenocarcinoma CRL5800, osteogenic sarcoma CRL1547, human breast cancer cell line MDA-MB-549, human neuroblastoma cell line SK-N-BE and human melanoma PLA-OD were examined as described in Figure 2. Results are mean ± SEM (*n* = 3 independent experiments). Asterisks indicate statistical significance compared to the untreated PBMC control values at 10 or 20% concentrations.

Having shown that supernatants from both egcSEs- and SEA-stimulated PBMCs were cytotoxic for Hep2 squamous cell carcinoma cells, we determined whether the cytotoxicity of these supernatants could also be demonstrated in broad sampling of the major human tumor histotologic types which included lung and breast carcinoma, melanoma, neuroblastoma, and osteogenic sarcoma cells. All egcSEs and SEA supernatants were equally effective in inducing significant concentration dependent cytotoxicity against all five human tumor cell lines compared to the unstimulated control (*p* < 0.005) (Figure 3). A hierarchy of sensitivity of the tumors to the cytotoxicity of the SE-stimulated PBMC supernatants is as follows: lung carcinoma > osteogenic sarcoma > melanoma > breast carcinoma > neuroblastona (Figure 3).

By contrast, we did not observe any direct toxic effect of 1 pg to 10 ng/mL of SEA and egcSE's on any the tumor cell lines tested herein (data not shown). To determine how tumor cell lines died upon addition of SE-activated PBMC supernatants, Annexin V, and PI staining was performed on Hep-2 tumor cells treated with 10% supernatants from SEA and egcSE-stimulated PBMCs. The percentage of annexinV^+^ cells was 10–45 fold higher than the percentage of IP^+^ annexinV^−^ cells at levels identical to those observed with unstimulated PBMC supernatants. These results suggest that upon addition of SE activated PBMC supernatants, cell lines died mainly by apoptosis.

### Nitrous oxide generation from egcSE-activated PBMCs

**Figure 4:**
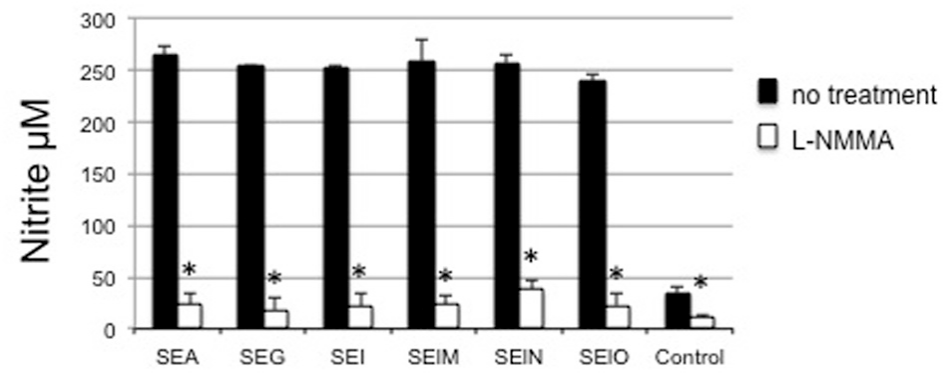
**Nitrite generation from SEs-stimulated PBMCs**. NO was quantified with Griess reagent in the supernatant of PBMCs (10^6^ cells/mL) incubated for 24 h in a presence of EMEM, SEA, SEG, SEI, SElM, SElN, or SElO, with or without NOS inhibitor, L-NMMA (300 μM). The results are mean ± SD (*n* = 3 independent experiments). Asterisks indicate statistical significance compared to values obtained without the NOS inhibitor.

Next we determined whether NO could be induced by egcSE-activated PBMCs and mediate a tumoricidal response. PBMCs stimulation by egcSEs and SEA was associated with a significant increase in nitrite production (*p* < 0.001) with no difference between toxins (*p* > 0.05) (Figure 4). As expected, the addition of nitrous oxide synthase inhibitor L-NMMA to PBMCs inhibited NO induction by all toxins (*p* < 0.001) with no significant differences in the degree of inhibition.

### Nitrous oxide synthase inhibition or neutralizing anti-TNF-α individually or combined reduce(s) tumor cell cytotoxicity of supernatants from egcSE-stimulated PBMCs

**Figure 5:**
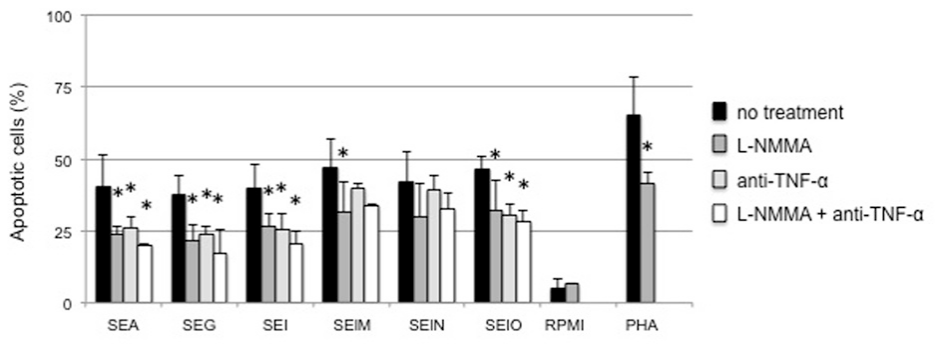
**Anti- TNF-α and NO inhibitor, alone and in combination inhibit Hep-2 tumor cells cytotoxicity induced by 10% supernatants from SEA and egcSE-stimulated PBMCs**. Hep-2 tumor cells cytotoxicity induced by 10% supernatants from SEA and egcSE-stimulated PBMCs before (black) or after treatment with L-NMMA (dark gray) or anti-TNF-α antibody (light gray) or a combination of L-NMMA plus anti-TNF-α antibody was analyzed by flow cytomerter using FITC-conjugated annexin-V and propidium iodide (IP) staining. The results are expressed as the mean ± SEM (*n* = 3 independent experiments). Asterisks indicate statistical significance compared to values obtained before treatment with L-NMMA or anti-TNF-α antibody.

Having shown that both egcSEs and SEA induced NO production by PBMCs, we determined whether the tumor cell cytotoxicity of the supernatants could be attenuated by the addition of nitrous oxide synthase inhibitor L-NMMA. Supernatants from 10% egcSE- and SEA-stimulated PBMCs induced tumor cell cytotoxicity. In all cases, tumor cell apoptosis was confirmed by annexin V-FITC/PI staining (Figure 5). Exposure of PBMCs to L-NMMA, a competitive inhibitor of NOS, before incubation with egcSEs or SEA induced a significant decrease in tumor cell cytotoxicity of all supernatants (range: *p* = 0.01 for SEA to *p* = 0001 for SEO) with the sole exception of SEN (*p* = 0.85) (Figure 5). Notably, supernatants from SEG and SEO-stimulated PBMCs showed more significant reductions in tumor cytotoxicity (*p* = 0.002 and *p* = 0.001, respectively) than SEA supernatants (*p* = 0.01) (Figure 5). We further determined whether tumor cell cytotoxicity of all SE supernatants could be attenuated by the addition of anti-TNF-α to L-NMMA. The combined treatments significantly reduced the cytotoxicity of SEA (*p* = 0.005) and three of the 5 egcSEs, namely SEG (*p* = 0.009), SEI (*p* = 0.013) and SEO (*p* = 0.001) (Figure 5). Notably, the inhibitory effects of L-NMMA and anti-TNF-α did not completely abolish the tumor cell cytotoxicity suggesting that additional tumoricidal factors are operative in this system.

### Cytokine profiles of supernatants induced by SE-activated PBMCs

Levels of cytokines TNF-α, IFN-y, IL-2, IL-4, IL-10, IL-17, and GM-CSF produced after stimulation of PBMCs from six healthy donors by egcSEs and SEA were measured. The absolute cytokine levels induced by each egcSE and canonical SEA are shown in Table 1. Cytokine levels of egcSEs I, M, N, and SEA-activated PBMCs were not significantly different [except for IL-4 and IL-10 after stimulation with SEO (*p* > 0.05)]. SEA induced significantly higher levels of cytokines than SEG with mean cytokine values 5, 8, 2, 6, 7, and 2 fold higher than SEG for GM-CSF, INF-γ, IL-2, IL-4, IL-10, IL-17, and TNFα, respectively (range *p* = 0.01–0.04, except for TNF-α).

**Table 1:** **Level of cytokines in supernatants of PBMC stimulated by SEA, SEG, SEI, SElM, SElN, and SElO**.

	**Cytokines (mean ± SEM)**						
	**GM-CSF**	**INF-γ**	**IL-2**	**IL-4**	**IL-10**	**IL-17**	**TNF-α**
SEA	474 ± 148	1816 ± 421	1210 ± 346	46 ± 8	674 ± 121	98 ± 19	852 ± 217
SEG	91 ± 33^*^	102 ± 38^*^	137 ± 43^*^	7 ± 2^*^	51 ± 15^*^	14 ± 4^*^	437 ± 162
SEI	652 ± 198	3344 ± 481	1354 ± 328	43 ± 5	366 ± 43	103 ± 20	1189 ± 262
SElM	282 ± 90	1249 ± 331	853 ± 226	18 ± 2	145 ± 17	50 ± 10	637 ± 199
SElN	290 ± 98	965 ± 326	826 ± 217	17 ± 3	232 ± 60	69 ± 22	692 ± 248
SElO	112 ± 37	888 ± 330	251 ± 33	8 ± 1^*^	76 ± 19^*^	41 ± 13	590 ± 217

Results are shown as the mean ± SEM for each point (n = 7 experiments with different blood donors). Asterisks indicate statistical significance compared to SEA.

## Discussion

Our results demonstrate that egcSEs activate 11–21% or resting T cells and that cell free supernatants (CFSs) from egcSE-stimulated PBMCs induced NO synthase activation and robust generation of NO along with TH-I TH-2 cytokines. Such CFSs from all egcSEs induced an equal degree of annexin positive apoptosis in a broad panel of clinically relevant human tumor cells with a hierarchy of sensitivity: lung carcinoma > osteogenic sarcoma > melanoma > breast carcinoma > neuroblastoma. The apoptotic effect of the egcSE CSFs appears to be mediated in part by NO and TNF-α since NO synthase inhibitor L-NMMA and anti-TNF-α antibodies significantly inhibited the tumor cell cytotoxicity. Moreover, all egcSE CFSs with the exception of SEG contained substantial levels of additional TH-1 cytokines such as IFN-γ that could contribute to the tumor cell cytotoxicity.

Hibbs et al., was the first to demonstrate that NO could inhibit tumor cell growth and/or induce tumor cell death by activated macrophages (Hibbs et al., 1987). Subsequently, NO-mediated tumor cell cytotoxicity has been demonstrated by a variety of immune cells including natural killer cells, T-cells, and endothelial cells (Albina and Reichner, 1998). Fast et al. showed that SEB and TSST-1-induced NO and TNF-α derived from activated macrophages could promote cytotoxicity of murine fibrosarcoma and mastocytoma cells (Fast et al., 1991). Herein, we extend these findings by showing that NO from egcSE-stimulated PBMCs contribute to the cytotoxicity of human tumor cells.

NO donors such as L-arginine from both endogenous and exogenous sources have been shown to exert an inhibitory action on the proliferation of tumor cells, such as breast cancer, mastocytoma, neuroblastoma, epidermoid carcinoma, pheochromocytoma, colon carcinoma, and pancreatic carcinoma cells *in vitro* (Maragos et al., 1993; Estrada et al., 1997; Buga et al., 1998; Gansauge et al., 1998; Pervin et al., 2001; Murillo-Carretero et al., 2002; Ruano et al., 2003; Tesei et al., 2003; Ciani et al., 2004; Huguenin et al., 2004; Bal-Price et al., 2006). The stimulatory or inhibiting behavior of NO appears to be related to the distinct concentrations of NO attained under different experimental conditions. For instance, iNOS-generated NO present in excess of 300 nmol/L promotes DNA damage, gene mutation and apoptosis via increased phosphorylation of p53 and expression of MKP-1, inhibition of phosphorylation of protein kinase C (PKC), extracellular-signal-regulated protein kinase (ERK) and JUN (Pervin et al., 2003; Jones et al., 2004; Thomas et al., 2004; Ridnour et al., 2005; Fukumura et al., 2006). NO donors have been shown to induce apoptotic cell death by inhibiting NF-κ B by phosphorylation of p50 via S-nitrosylation (Marshall and Stamler, 2001), binding to iron-sulfur centers and inhibiting aconitase, complex I/complex II of the mitochondrial respiratory chain, or ribonucleotide reductase (Stuehr and Nathan, 1989; Lepoivre et al., 1990). In lower concentrations, tumor cell NO promotes tumor growth, neovascularization and invasiveness by induction of p53 mutations, upregulation of vascular endothelial growth factor resulting in neovascularization, increased vascular permeability and vasodilatation (Krischel et al., 1998; Ulibarri et al., 1999; Frank et al., 2000; Luczak et al., 2004; Wai et al., 2006). In the present study, a low dose of each egcSE induced robust nitrite concentrations of 200–250 μM suggesting that these agents may be capable of inducing a sufficient quantity of NO to exert a tumoricidal effect *in vivo*.

*In vivo*, exogenous NO or endothelial-cell derived NO in higher concentrations appears to present a tumoricidal barrier inimical to tumor cell dissemination. Selective genetic or pharmacological inhibition of eNOS or iNOS or enzymatic induction of NO deficiency in tumor cells diminishes VEGF, HO-1, and HIF1α activation and consequent tumor cell proliferation and angiogenesis (Kimura et al., 2000; Motterlini et al., 2000; Naughton et al., 2002; Kasuno, 2004). Local release of NO in endothelial cells, liver sinusoids, or pulmonary circulation causes apoptosis of the disseminated tumor cells at these sites (Fukumura et al., 1996; Wang et al., 2000, 2007; Qiu et al., 2003; Qi et al., 2004). Finally, daily intraperitoneal injections of NO-producing nitrovasodilators isosorbide mono-and dinitrate resulted in a significant decrease of the size of the primary tumor and a reduction in the number and size of spontaneous lung metastases (Pipili-Synetos et al., 1995). Thus, NO donors delivered parenterally or released from activated endothelial cells in sufficient concentration appears to be capable of inducing local tumor cell death and limiting tumor metastases.

In our study, NO and TNF-α in the CSF exhibited an additive effect in tumor cell cytotoxicity. Indeed, all of the egcSEs induced robust levels of NO and TNF-α with the exception of SEG as discussed below. TNF-α has direct effects on a variety of cell types and has been shown to work together with NO in tumor cell cytotoxicity (Laster et al., 1988; Estrada et al., 1992). NO is known to sensitize tumor cells to TNF-α-mediated apoptosis via specific disruption of the TNF-α-induced generation of hydrogen peroxide and subsequent inhibition of the NF-κ B dependent expression of anti-apoptotic genes (Schreck et al., 1992; Hong et al., 1997). Moreover, G1 arrest has been attributed to endogenous NO following activation by TNF-α, IFN-γ and IL-1 in breast and pancreatic carcinoma cells (Gansauge et al., 1998; Pervin et al., 2001). Furthermore, all of the egcSEs except SEG were also shown herein to be potent inducers of IFN-γ that could contribute to the tumor cytotoxic response.

In addition to its tumor killing properties, TNF-α has also been identified as the major cause of SE-induced toxicity in mice (Bette et al., 1993; Miethke et al., 1993). In previous cancer trials, the systemic toxicity of SEA was presumed to be related to TNF-α (Giantonio et al., 1997; Cheng et al., 2004). As shown herein, while SEG induced robust production of nitrite from PBMCs and robust tumor cell cytotoxicity, it also displayed substantially lower quantities of TNF-α and IFN-γ relative to other egcSEs. Interestingly, a supernatant from *S. aureus* producing SEG together with the other egcSEs used in a recent cancer trial exhibited minimal systemic toxicity (Ren et al., 2004). SEG's potent T cell activation (comparable to SEA) coupled with its significantly reduced cytokine levels (relative to SEA and the other egcSEs) is reminiscent of split T cell responses when classic peptide antigen is presented to T cells in the absence B7 costimulation (Schweitzer and Sharpe, 1998). In this context, wild type SEA has been shown to possesses an intirisic costimulatory B7-like sequence in its conserved β-strand/hinge/α-helix domains that engages and activates the T cell costimulatory CD28 homodimer resulting in T cell cytokine secretion; mutation of this sequence resulted in attenuated cytokine production (Arad et al., 2011). SEG possesses several amino acid substitutions in this conserved sequence which could alter its topographic interface with T cell CD28 resulting in reduction in cytokine levels noted herein. These findings suggest that the nature of SEG's T cell response depends not only on its affinity for MHCII but also on the strength of its intrinsic costimulatory interaction with T cell CD28. SEG's retention of NO-dependent tumor cell cytotoxicity and robust T cell activation in the presence of low cytokine levels makes it a promising model for such investigation.

Neutralizing antibodies against superantigens are considered to be the major factors underlying superantigen therapy failure. It was recently confirmed in the completed Phase II/III trial using a hybrid SEA/SEE120 superantigen (fused to a mouse monoclonal Fab targeting the 5T4 antigen on tumor cells) that SEA/SEE120 did not improve overall survival in advanced renal cell cancer (Hawkins et al., 2013). The ineffectiveness of this drug was likely due to the higher than expected levels of pre-existing anti-SEA/SEE120 antibodies because a small subgroup of patients with low levels of anti-SEA/SEE120 antibodies did apparently demonstrate longer survival. Importantly, prevalence of antibodies against egcSEs including SEG is much lower than antibodies against classical SEs (Holtfreter et al., 2004). Our results demonstrating SEG's retention of NO-dependent tumor cell cytotoxicity and robust T cell activation in the presence of low cytokine levels suggests SEG may be more appropriate for superantigen therapy in cancer.

### Conflict of interest statement

The authors declare that the research was conducted in the absence of any commercial or financial relationships that could be construed as a potential conflict of interest.
